# Monitored Anesthesia Care of Two Patients with Highly Elevated Subpulmonic Ventricular Pressure due to Adult Congenital Heart Disease

**DOI:** 10.1155/2020/2040561

**Published:** 2020-01-11

**Authors:** Tatsuya Kida, Tomoya Irie, Takahisa Goto

**Affiliations:** ^1^Department of Anesthesiology, Yokohama City University School of Medicine, 3-9 Fukuura, Kanazawa-ku, Yokohama 2360004, Japan; ^2^Department of Anesthesiology, Yokohama City University Medical Center, 4-57 Urahune, Minami-ku, Yokohama 2320024, Japan

## Abstract

Procedural sedation and analgesia for patients with adult congenital heart disease (ACHD) and highly elevated subpulmonic ventricular pressure require proper anesthesia care to prevent a pulmonary hypertensive crisis. We describe the monitored anesthesia care (MAC) of two patients with ACHD (a complete atrioventricular septal defect and congenitally corrected transposition of the great arteries) and highly elevated subpulmonic ventricular pressure. In both patients, preprocedural transthoracic echocardiography was useful for detecting severely elevated subpulmonic ventricular pressure. The MAC involved the infusion of propofol, dexmedetomidine, and fentanyl. Norepinephrine was continuously administered from the preanesthetic period. No hemodynamic instability or respiratory depression was observed during the MAC. Continuous administration of norepinephrine from the preinduction period was helpful for preventing hypotension. We added dexmedetomidine to our MAC regimen of propofol and fentanyl because it exerts both sedative and analgesic effects. Dexmedetomidine does not cause respiratory depression; thus, our MAC regimen is believed to be theoretically safe for patients with ACHD and elevated subpulmonic ventricular pressure. Our findings suggest that safe MAC for patients with ACHD and highly elevated subpulmonic ventricular pressure may require careful consideration of the anesthetic regimen and close observation by adequately trained personnel, which is best provided at regional ACHD centers.

## 1. Background

**Figure 1:**
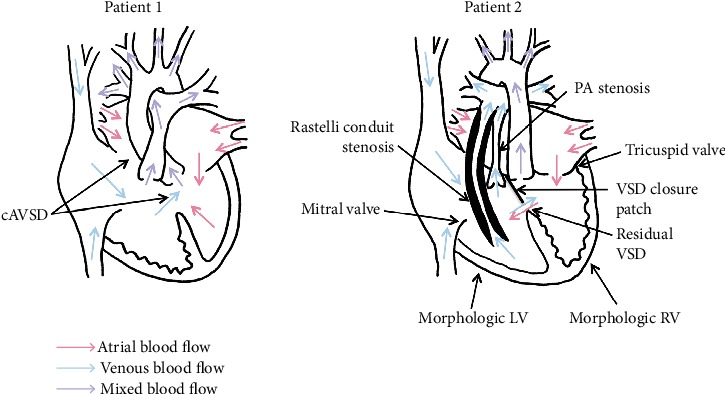
Illustrations of the cardiac anatomy and physiology of two patients with adult congenital heart disease (ACHD) and highly elevated ventricular pressure circulating to the pulmonary artery (PA). cAVSD, complete atrioventricular septal defect; PA: pulmonary artery; VSD: ventricular septal defect; LV: left ventricle; RV: right ventricle.

The population of patients with adult congenital heart disease (ACHD) has been increasing in recent years [1–3]. Therefore, physicians may encounter such patients requiring invasive procedures in their daily clinical practice. While monitored anesthesia care (MAC) provides good procedural conditions, it presents a great challenge to anesthesia providers. However, the literature is scarce with respect to the risks in patients with ACHD and highly elevated subpulmonic ventricular pressure undergoing MAC [4, 5]. Inadequate sedation and analgesia may cause an abrupt increase in pulmonary vascular resistance (PVR) in response to procedural stimuli. On the other hand, excessive use of anesthetic and analgesic agents may cause cardiorespiratory depression, which worsens pulmonary arterial pressure, causing subpulmonic ventricular failure. Thus, patients with ACHD and elevated subpulmonic ventricular pressure have a very narrow margin of safety with regard to sedation and analgesia. In this report, we describe the successful MAC of two patients with ACHD and highly elevated subpulmonic ventricular pressure.  Figure 1 provides a schematic demonstration of the circulation in each patient. The authors confirm that written informed consent for submission and publication of this report has been obtained from both patients.

## 2. Case Presentation

### 2.1. Patient 1

**Figure 2:**
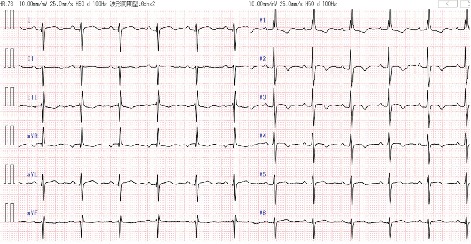
The preprocedural electrocardiogram of patient 1 shows sinus rhythm, northwest axis, incomplete right bundle branch block pattern, and biphasic QRS complexes in V2-5 and deep *S* waves in V5-6.

A 22-year-old woman with Down syndrome and Eisenmenger syndrome due to a complete atrioventricular septal defect (cAVSD) that was diagnosed at birth was scheduled for central venous catheter (CVC) placement for acute myelocytic leukemia management. The patient was prescribed tadalafil and furosemide, and her condition was classified as New York Heart Association (NYHA) class II. The preprocedural brain natriuretic peptide (BNP) level was 22.6 pg/mL. Her electrocardiogram (ECG) showed sinus rhythm, northwest axis, incomplete right bundle branch block pattern, and biphasic QRS complexes in V2-5 and deep S waves in V5-6 (Figure 2). Transthoracic echocardiography (TTE) showed mild common atrioventricular valve regurgitation and persistently elevated right ventricular pressure (RVP), which was estimated to be equal to or higher than the left ventricular pressure (LVP) indicated by interventricular septal (IVS) motion. Right ventricular function was preserved with a tricuspid annular plane systolic excursion (TAPSE) of 23.5 mm and *E*/*A* 0.94; *E*/*e*′ 4.9. Left ventricular function was also preserved with a fraction area change (FAC) of 56% and *E*/*A* 0.97; *E*/*e*′ (septal) 20; *E*/*e*′ (lateral) 11. Since central venous catheter placement is an invasive procedure, MAC or general anesthesia with tracheal intubation (GETA) was required in this patient to avoid sudden increases in PVR because of noncooperation, crying, screaming, or struggling. Since MAC is less invasive than GETA, we scheduled the patient for CVC placement under MAC.

**Figure 3:**
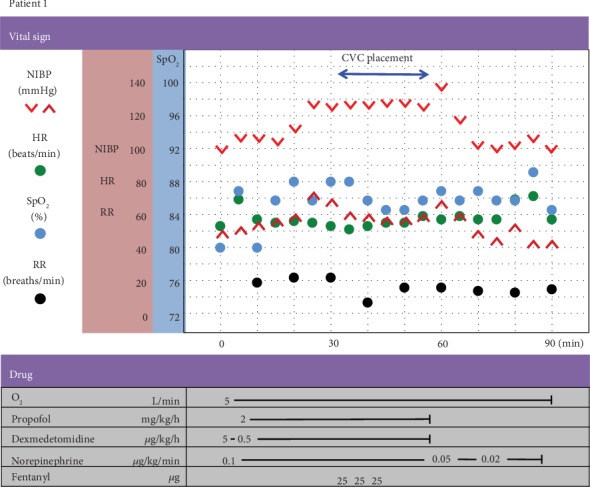
Anesthesia record of patient 1. NIBP: noninvasive blood pressure; HR: heart rate; SpO_2_: oxygen saturation measured by pulse oximetry; RR: respiratory rate; CVC: central venous catheter.

The anesthesia record is shown in Figure 3. Her preprocedural vital signs in the operating room were as follows: blood pressure (BP)—98/52 mmHg; heart rate (HR)—54 beats/min; and oxygen saturation measured by pulse oximetry (SpO_2_)—80% in room air. During MAC, 5 L/min of 100% oxygen was administered via face mask. End-tidal carbon dioxide (EtCO_2_) was monitored with capnography. Before the induction of anesthesia, a continuous peripheral venous infusion of norepinephrine was initiated at 0.1 *μ*g/kg/min and maintained at 0.02–0.1 *μ*g/kg/min to maintain the systolic systemic arterial blood pressure (sSABP) above 100 mmHg (her resting sSABP). MAC was provided with a continuous infusion of propofol (2 mg/kg/h), dexmedetomidine (a loading dose of 1 *μ*g/kg over 10 minutes followed by a 0.5 *μ*g/kg/h infusion), and fentanyl (total dose: 75 *μ*g) to achieve optimal sedation and analgesia. Local anesthesia was obtained by a superficial injection of 1% lidocaine. During MAC, glossoptosis occurred temporarily and was easily rectified by a jaw lift. The CVC was placed successfully without hemodynamic instability, respiratory depression, or oxygen desaturation. The infusion of norepinephrine was tapered according to sSABP and discontinued after 30 minutes of the procedure. The level of consciousness was arousable on calling, and no airway obstruction was observed at the end of the MAC. The postprocedural course was uneventful. Chemotherapy was started on postprocedural day 4.

### 2.2. Patient 2

**Figure 4:**
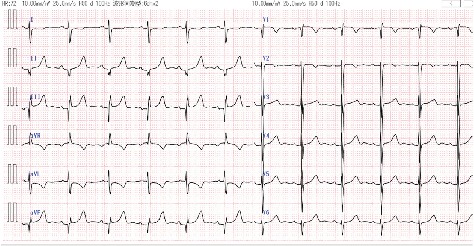
The preprocedural electrocardiogram of patient 2 shows sinus rhythm, northwest axis, notched *P* wave, and Qr pattern in V1.

A 39-year-old woman presented for transesophageal echocardiography (TEE) to rule out intracardiac thrombus. She had chronic cough and progressive dyspnea, and a history of a ventricular septal defect (VSD), pulmonary artery (PA) stenosis, and congenitally corrected transposition of the great arteries (ccTGA). At 8 years of age, she underwent the Rastelli procedure only, which connected the morphologic left ventricle (LV) to the PA via a conduit graft for PA stenosis. She did not undergo anatomic repair of the corrected transposition, in which an atrial inversion procedure (Senning or Mustard) is combined with either an arterial (arterial switch) or ventricular (Rastelli procedure) level repair. The patient was not prescribed any medications and was NYHA class II. The preprocedural BNP level was 50.1 pg/mL. The ECG showed sinus rhythm, northwest axis, notched *P* wave, and Qr pattern in V1 (Figure 4). TTE showed persistently elevated morphologic LVP, which was estimated to be equal to morphologic RVP. The suspected morphologic LV volume overload was indicated by a residual VSD with bidirectional shunting (diameter: 18 mm), mild mitral regurgitation, mild tricuspid regurgitation, and a suspected thrombus in the right atrium. Morphologic right ventricle (RV) function was preserved with an ejection fraction (EF) of 61% and *E*/*A* 1.59; *E*/*e*′ 12.4. Morphologic LV function was also preserved with an FAC of 49%, mitral annular plane systolic excursion (MAPSE) of 20.9 mm, and *E*/*A* 1.91; *E*/*e*′ (septal) 11; *E*/*e*′ (lateral) 10. Although TTE could not detect the Rastelli conduit, progression of conduit stenosis was suspected from her clinical course and other echocardiography data. She was scheduled for TEE under MAC for a detailed observation of the thrombus.

**Figure 5:**
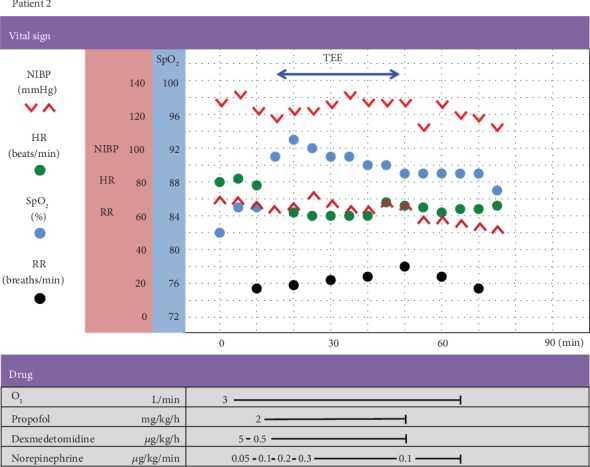
Anesthesia record of patient 2. NIBP: noninvasive blood pressure; HR: heart rate; SpO_2_: oxygen saturation measured by pulse oximetry; RR: respiratory rate; TEE: transesophageal echocardiography.

The anesthesia record is shown in Figure 5. Her preprocedural vital signs in the operating room were as follows: BP—126/72 mmHg; HR—80 beats/min; and SpO_2_—82% in room air. During MAC, 3 L/min of 100% oxygen was administered via a nasal cannula. Her EtCO_2_ was monitored with capnography. Before the induction of anesthesia, a continuous peripheral venous infusion of norepinephrine was initiated at 0.05 *μ*g/kg/min and maintained at 0.05–0.3 *μ*g/kg/min to maintain the sSABP above 110 mmHg (her resting sSABP). MAC was provided with a continuous infusion of propofol (2–2.3 mg/kg/h) and dexmedetomidine (a loading dose of 1 *μ*g/kg over 10 minutes followed by a 0.5 *μ*g/kg/h infusion) to achieve optimal sedation and analgesia. For local anesthesia, 2% viscous lidocaine was used on the pharynx and larynx. No hemodynamic instability, respiratory depression, or oxygen desaturation was observed during MAC. The infusion of norepinephrine was tapered according to sSABP and discontinued after 20 minutes of the procedure. The level of consciousness was arousable on calling, and no airway obstruction was observed at the end of the MAC. The postprocedural course was uneventful. The patient was discharged on postprocedural day 1.

## 3. Discussion

We successfully provided MAC to two patients with ACHD and elevated subpulmonic ventricular pressure with an intracardiac shunt. The clinical courses of these two patients suggest several important points for anesthesia providers.

First, we found that a continuous infusion of norepinephrine from the preinduction period was helpful in maintaining hemodynamic stability in these patients. Many agents commonly used for sedation and anesthesia, such as propofol, decrease the systemic vascular resistance (SVR), which may increase the right-to-left shunt flow and cause oxygen desaturation [6, 7]. Moreover, perfusion of the coronary artery that supplies the subpulmonic ventricle is dependent on a pressure gradient between the aorta and the subpulmonic ventricle. When the subpulmonic ventricular pressure is elevated, decreased SVR reduces coronary blood flow to the subpulmonic ventricle for pulmonary circulation, leading to subpulmonic ventricular dysfunction. Furthermore, the morphologic RV was a ventricle for systemic circulation in patient 2. In ACHD patients with ccTGA that have not undergone anatomic repair of the corrected transposition, the morphological RV is forced to generate the pressure required to overcome the systemic afterload. However, the morphologic RV is not anatomically suited to withstand the systemic pressure, unlike the morphologic LV. Therefore, increased or decreased SVR directly correlates with deterioration of morphologic RV systolic function, which causes a further decrease in the cardiac output. A titrated infusion of vasopressors from the preinduction period effectively counteracts anesthesia-induced vasodilation. According to a review of 33 patients with Eisenmenger syndrome or a similar pathology, the use of a vasopressor significantly decreased the incidence of hypotension during anesthetic induction [8]. In both of our patients, norepinephrine was peripherally infused before anesthesia induction and titrated to maintain the sSABP above the resting level throughout the procedure. Other vasopressors such as phenylephrine, dopamine, and vasopressin may also be used to raise the sSABP. Norepinephrine is preferred over phenylephrine because of the *β*1 effect of the former, which increases ventricular contractility and helps counteract the increase in PVR. Dopamine titration is more complicated than that of norepinephrine because of the dose-dependent shift in the predominant effects of dopamine. Vasopressin is a nonsympathomimetic vasopressor, which has the possibility of decreasing PVR without decreasing SVR [9, 10]. However, because of its much longer half-life (10–30 min) than that of norepinephrine (1–2.5 min), vasopressin is difficult to titrate in response to rapid changes in the sSABP. A continuous basal infusion of vasopressin may be used as an adjunct to norepinephrine.

Second, an increase in PVR also should be prevented. Any catecholamine surge caused by pain, agitation, or anxiety will abruptly increase PVR, so judicious doses of sedatives and analgesics should be provided to prevent it. Propofol is an intravenous sedative drug that provides effective sedation and rapid recovery from anesthesia. Owing to its extensive usage in anesthesia patients, we chose propofol as the first-choice sedative. Fentanyl is a commonly used analgesic for MAC, but may cause dose-dependent hypercarbia, which may increase PVR. To avoid this problem, we used local anesthetics and also added dexmedetomidine to our anesthetic regimen. Dexmedetomidine is a selective *α*2 adrenergic agonist with sedative, analgesic, and anxiolytic properties but does not promote respiratory depression [11]. Dexmedetomidine may help to reduce the propofol and fentanyl requirements and thereby may lower the risk of hypercarbia [12, 13]. A pilot study of 22 pediatric patients following congenital heart disease surgery demonstrated that dexmedetomidine did not increase pulmonary arterial pressure [14]. We did not observe substantial respiratory depression in either of our cases as indicated by a reduced respiratory rate and/or elevated EtCO_2_ compared with baseline levels.

Third, continuous and careful observation of airway patency is of paramount importance during MAC. We chose MAC with spontaneous breathing over general anesthesia with endotracheal intubation and positive pressure ventilation because we believe that the former might be more advantageous in lowering PVR compared with the latter. However, once the airway is compromised, hypoxia may ensue and increase PVR. Therefore, in both cases, we always had at least one designated anesthesiologist at the bedside who continuously monitored the EtCO_2_ and other clinical signs of adequate airway patency who could intervene if airway obstruction occurred.

Procedural sedation and analgesia without careful consideration of the anesthetic regimen can be harmful to patients with ACHD and highly elevated subpulmonic ventricular pressure. In fact, we suppose that these patients have periprocedural risks similar to those observed in patients with severe pulmonary arterial hypertension [15]. There are many sedative and analgesic drug choices for MAC [16]. However, there is no conclusive evidence for the most effective drug. Although MAC with a combination of propofol, dexmedetomidine, and fentanyl provides safe and effective sedation and analgesia, drug interactions among these three anesthetics complicate anesthetic management. Patients with ACHD and highly elevated subpulmonic ventricular pressure may receive better anesthetic care at an appropriately equipped regional ACHD center with an experienced cardiac anesthesiologist.

## 4. Conclusion

In the anesthetic management of patients with ACHD and highly elevated subpulmonic ventricular pressure, it is most important to prevent a pulmonary hypertensive crisis. Most anesthetic agents have a hypotensive effect, which causes poor coronary perfusion, right-to-left shunting, and subpulmonic ventricle dilation/failure. Continuous administration of adequate norepinephrine from during the preanesthetic period is crucial for the prevention of sudden systemic hypotension. Although MAC with a combination of propofol, dexmedetomidine, and fentanyl provides safe and effective sedation and analgesia, it may be better performed by a trained anesthesiologist. Patients with ACHD and highly elevated subpulmonic ventricular pressure may be treated at a regional ACHD center.
